# High-yield anaerobic succinate production by strategically regulating multiple metabolic pathways based on stoichiometric maximum in *Escherichia coli*

**DOI:** 10.1186/s12934-016-0536-1

**Published:** 2016-08-12

**Authors:** Jiao Meng, Baiyun Wang, Dingyu Liu, Tao Chen, Zhiwen Wang, Xueming Zhao

**Affiliations:** 1 Department of Biochemical Engineering, School of Chemical Engineering and Technology, Tianjin University, Tianjin, 300072 People’s Republic of China; 2 Key Laboratory of Systems Bioengineering (Ministry of Education), Tianjin University, Tianjin, People’s Republic of China; 3 SynBio Research Platform, Collaborative Innovation Center of Chemical Science and Engineering (Tianjin), School of Chemical Engineering and Technology, Tianjin University, Tianjin, People’s Republic of China; 4 Key Laboratory of Fermentation Engineering (Ministry of Education), Hubei Provincial Cooperative Innovation Center of Industrial Fermentation, Hubei University of Technology, Wuhan, 430068 People’s Republic of China

**Keywords:** *Escherichia coli*, Succinate, Anaerobic, Pentose phosphate pathway, Transhydrogenase, Cofactor

## Abstract

**Background:**

Succinate has been identified by the U.S. Department of Energy as one of the top 12 building block chemicals, which can be used as a specialty chemical in the agricultural, food, and pharmaceutical industries. *Escherichia coli* are now one of the most important succinate producing candidates. However, the stoichiometric maximum succinate yield under anaerobic conditions through the reductive branch of the TCA cycle is restricted by NADH supply in *E. coli*.

**Results:**

In the present work, we report a rational approach to increase succinate yield by regulating NADH supply via pentose phosphate (PP) pathway and enhancing flux towards succinate. The deregulated genes *zwf243* (encoding glucose-6-phosphate dehydrogenase) and *gnd361* (encoding 6-phosphogluconate dehydrogenase) involved in NADPH generation from *Corynebacterium glutamicum* were firstly introduced into *E. coli* for succinate production. Co-expression of beneficial mutated dehydrogenases, which removed feedback inhibition in the oxidative part of the PP pathway, increased succinate yield from 1.01 to 1.16 mol/mol glucose. Three critical genes, *pgl* (encoding 6-phosphogluconolactonase), *tktA* (encoding transketolase) and *talB* (encoding transaldolase) were then overexpressed to redirect more carbon flux towards PP pathway and further improved succinate yield to 1.21 mol/mol glucose. Furthermore, introducing *Actinobacillus succinogenes pepck* (encoding phosphoenolpyruvate carboxykinase) together with overexpressing *sthA* (encoding soluble transhydrogenase), further increased succinate yield to 1.31 mol/mol glucose. In addition, removing byproduct formation through inactivating acetate formation genes *ackA*-*pta* and heterogenously expressing *pyc* (encoding pyruvate carboxylase) from *C. glutamicum* led to improved succinate yield to 1.4 mol/mol glucose. Finally, synchronously overexpressing *dcuB* and *dcuC* encoding succinate exporters enhanced succinate yield to 1.54 mol/mol glucose, representing 52 % increase relative to the parent strain and amounting to 90 % of the strain-specific stoichiometric maximum (1.714 mol/mol glucose).

**Conclusions:**

It’s the first time to rationally regulate pentose phosphate pathway to improve NADH supply for succinate synthesis in *E. coli*. 90 % of stoichiometric maximum succinate yield was achieved by combining further flux increase towards succinate and engineering its export. Regulation of NADH supply via PP pathway is therefore recommended for the production of products that are NADH-demanding in *E. coli*.

**Electronic supplementary material:**

The online version of this article (doi:10.1186/s12934-016-0536-1) contains supplementary material, which is available to authorized users.

## Background

Succinate and its derivatives are widely used as specialty chemicals in the field of food, pharmaceutical, and cosmetic industries [1]. The industrial potential for succinate fermentations was recognized as early as 1980 [2]. Several bacteria, such as, *Anaerobiospirillum succiniciproducens* [3], *Actinobacillus succinogenes* [4], and *Mannheimia succiniciproducens* [5], have been isolated and manipulated for succinate production. However, those rumen organisms usually require high-cost nutrient sources for growth and perform characteristically unstable in the process of fermentation. Recent studies on metabolic engineering of *Escherichia coli* for succinate production have been carried out due to the ease of genetic manipulation coupled to its rapid growth rate, standardized cultivation techniques, and inexpensive media.

In anaerobic conditions, *E. coli* proceeds with mixed-acid fermentation but succinate represents a minor product [6]. Derivatives of *E. coli* have been constructed in efforts to improve succinate production with variable success. Most genetic engineering strategies focused on eliminating competing pathways [7–9], overexpressing native or heterologous key enzymes [9–12], disrupting phosphotransferase (PTS) system to improve PEP supply [12, 13], activating glyoxylate pathway [14, 15], and combining metabolic engineering and metabolic evolution [11, 16, 17]. Some other external means, such as a dual phase fermentation production mode which comprises an initial aerobic growth phase followed by an anaerobic production phase, have also been developed in order to increase succinate production [18].

**Fig. 1:**
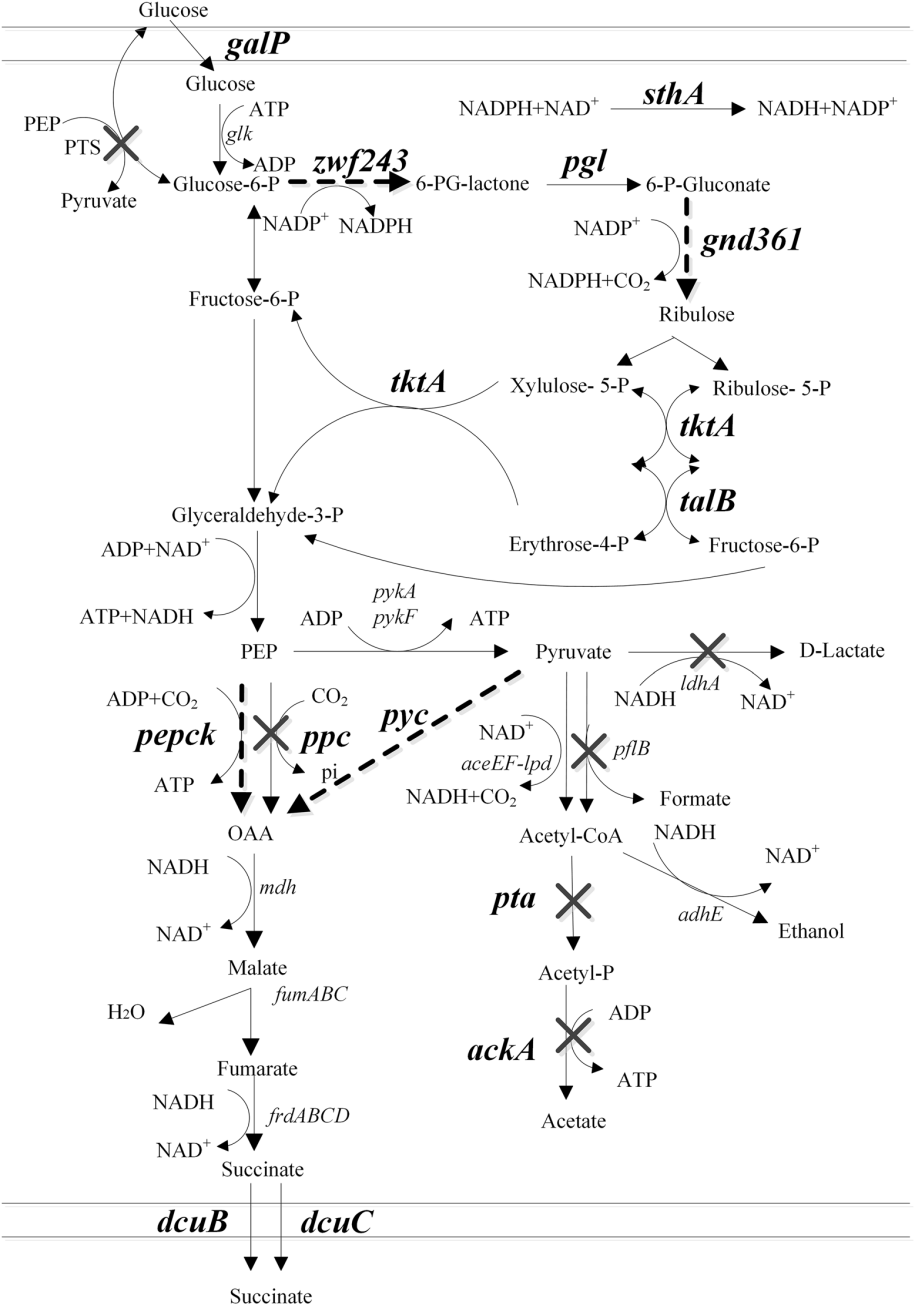
Metabolic network of *E. coli* under anaerobic conditions and metabolic engineering strategies for succinate overproduction. Modified genes for improving succinate yield are highlighted in *bold*. *X* indicates metabolic reactions that have been blocked by gene deletions. *Dotted arrows* indicate steps that are heterogeneously expressed in *E. coli.* Genes and enzymes: *ldhA* encoding lactate dehydrogenase; *pflB* encoding pyruvate-formate lyase; *pta* encoding phosphate acetyltransferase; *ackA* encoding acetate kinase; *adhE* encoding alcohol dehydrogenase; *ppc* encoding phosphoenolpyruvate carboxylase; *pepck* encoding phosphoenolpyruvate carboxykinase; *pyc* encoding pyruvate carboxylase; *aceEF*-*lpd* encoding pyruvate dehydrogenase complex; *mdh* encoding malate dehydrogenase; *fumA*, *fumB*, and *fumC* encoding fumarase isozymes; *frdABCD* encoding fumarate reductase; *galp* encoding galactose permease; *glk* encoding glucokinase; *PTS* phosphotransferase systems; *zwf243* encoding 6-phosphate dehydrogenase; *gnd361* encoding 6-phosphogluconate dehydrogenase; *pgl* encoding 6-phosphogluconolactonase; *tktA* encoding transketolase; *talB* encoding transaldolase; *sthA* encoding soluble transhydrogenase

In wild type *E. coli*, the reductive tricarboxylic acid (TCA) branch significantly contributed to anaerobic succinate production (Fig. 1). One major obstacle to high succinate yield through this fermentative pathway is NADH availability [14, 17]. Inactivating NADH competing pathways has often been applied to improve NADH availability for succinate production [19], while deletion of these genes that block the competing routes for NADH oxidation were insufficient to direct carbon flow to succinate [20]. Glycerol, as an alternative substrate to glucose, has become an appealing substrate for biological synthesis and was applied to succinate synthesis due to its high degree of reduction [21]. When glycerol was used as a carbon source in the anaerobic fermentation of *E. coli*, higher levels of NADH are generated in glycerol dissimilation through glycerol dehydrogenase (GldA) [21, 22]. Recently, the NAD^+^-dependent formate dehydrogenase from *Candida boidinii* was introduced into an *E. coli* strain [23]. The introduced formate dehydrogenase converted formate into NADH and CO_2_, which not only increased succinate productivity but reduced concentrations of byproduct formate. However, this strategy is usually associated with the carbon source loss or requires formate addition. In addition, the glyoxylate pathway can also form succinate, which is essentially active under aerobic condition, and conserves NADH consumption relative to reductive TCA branch in succinate formation [14, 15]. It has been reported that succinate yield can reach its maximum level 1.714 mol/mol glucose by redirecting 71.4 % of carbon flows to the reductive TCA branch and 28.6 % of the carbon flows to the glyoxylate pathway [10], while employment of the glyoxylate bypass for succinate synthesis is accompanied by the CO_2_ loss [24].

Pentose phosphate (PP) pathway was known to play major roles in NADPH generation and modulation of the PP pathway has been conventionally attempted as primary solution to increase NADPH-dependent biotransformation [25, 26]. In *E. coli*, there exist two types of pyridine nucleotide transhydrogenase which are responsible for the mutual transformation between NADPH and NADH [26, 27]. They are energy-dependent membrane-bound transhydrogenase (TH or PntAB, EC 1.6.1.2) and energy-independent soluble transhydrogenase (SthA or UdhA, EC 1.6.1.1). The membrane-bound PntAB catalyzes the transfer of a hydrogen ion from NADH to NADPH under low levels of NADPH, while the soluble SthA catalyzes the transfer of a hydrogen ion from NADPH to NADH under high concentrations of NADPH [26]. Recently, a high-succinate-producing strain HX024 was obtained through metabolic evolution. It was found that pyruvate dehydrogenase (PDH) was significantly activated, and sensitivity of PDH to NADH was eliminated by three mutations in LpdA, which is the E3 component of PDH. Another reasons for high yield of strain HX024 were ascribed to the increased transketolase and soluble transhydrogenase SthA activities [17]. It should be noted that PP pathway converts glucose-6-phosphate into fructose-6-phosphate and glyceraldehyde-3-phosphate with NADPH produced, the carbon flux from synthesized fructose-6-phosphate and glyceraldehyde-3-phosphate then go through glycolytic pathway and produce PEP and NADH, and NADPH could be converted into NADH by native transhydrogenase SthA for succinate synthesis. Therefore, it should be a potential strategy to regulate NADH supply for succinate synthesis by rationally modifying PP pathway in *E. coli*.

In the present work, a reducing equivalent-conserving pathway, which combined the coordinated re-engineering of the glycolytic pathway and the pentose phosphate pathway, was implemented for the first time to provide more NADH to achieve the stoichiometric maximum succinate yield 1.714 mol/mol glucose. NADH availability was then increased for succinate synthesis through modifying pentose phosphate pathway. Systems metabolic engineering was followed to further improve succinate yield, which involves in increasing carbon flux towards succinate, reinforcing NADH level, and finally engineering succinate export. As a result of these modifications, 90 % of stoichiometric maximum succinate yield was achieved. The strategy for improving NADH supply described here may be useful for engineering *E. coli* strains for the industrial production of succinate and related NADH-demanding products.

## Results

### Rationale for enhancing NADH supply by modifying pentose phosphate pathway

We presumed that succinate is formed exclusively via the reductive TCA branch. Glucose is internalized into the cytoplasm and then catalyzed into glucose-6P by glucokinase (encoded by *glk*), which was then catabolized into PEP and NADH for succinate synthesis. The cells are capable of producing PEP and NADH from glucose at two different modes.

The first mode corresponds to the formation of PEP and NADH exclusively via glycolytic pathway, the reaction may be expressed as:

1 $${\text{Glucose}} \to 2{\text{PEP}} + 2{\text{NADH }}$$

The second mode implies the involvement of pentose phosphate pathway in the PEP and NADH formation, the reaction may be expressed as:

2 $$\eqalign{ & {\text{Glucose}} \to 5/3{\text{PEP}} + 2{\text{NADPH}} + 5/3{\text{NADH}} \cr & \quad \qquad \qquad+ \,{\text{C}}{{\text{O}}_2} \cr}$$

NADPH could be converted into NADH by transhydrogenase SthA, the reaction may be expressed as:

3 $${\text{NADPH}} + {\text{NAD}}^{ + } \mathop \to \limits^{\text{SthA}} {\text{NADH }} + {\text{NADP}}^{ + }$$

Integrating of the reaction (2), (3), we can get the reaction:

4 $${\text{Glucose}} \to 5/3{\text{PEP}} + 11/3{\text{NADH}} + {\text{CO}}_{2}$$

PEP was reduced to become succinate via reductive TCA branch, in which two molar NADH were required. The reaction may be expressed as:

5 $${\text{PEP}} + {\text{CO}}_{2} + 2{\text{NADH}} \to {\text{succinate}}$$

It is not difficult to find that NADH is in excess for reducing PEP to from succinate via PP pathway in reaction (4), while NADH is insufficient for reducing PEP to from succinate in reaction (1). According to redox balance, the maximum stoichiometric succinate yield of 1.714 mol/mol glucose can be obtained if the carbon flux ratio between PP pathway and glycolysis pathway is 6:1.

### Succinate yield is effectively enhanced by redirection of flux towards pentose phosphate pathway

**Fig. 2:**
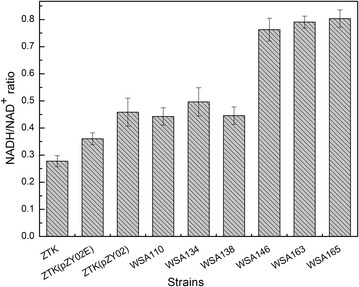
Changes in the levels of intracellular NADH/NAD^+^ ratio in different strains. Bacteria were cultivated with 50 mL NBS medium in a 100 mL flask shaking at 220 rpm and 37 °C in anaerobic conditions with an initial OD600 of 0.6. The intracellular NADH, NAD^+^ were extracted at exponential phase. Data are average values and standard deviations of triplicate experiments

As shown in Fig. 1, glucose-6-phosphate dehydrogenase (encoded by *zwf*) and 6-phosphogluconate dehydrogenase (encoded by *gnd*) are involved in NADPH generation in the oxidative part of the PP pathway, and many studies have focused on the modulation of these two endogenous enzymes to improve NADPH-dependent biotransformation in *E. coli* [26]. While both enzymes are inhibited by NADPH, ATP, fructose 1,6-bisphosphate (FBP) and the 6PGD additionally by D-glyceraldehyde 3-phosphate (Gra3P), erythrose 4-phosphate and Ru5P. The feedback regulation implies that PP pathway can respond very flexibly to altered NADPH and product levels. Overexpression of the deregulated genes *zwf243* (with point mutation A243T) and *gnd361* (with point mutation S361F), which led to release of feedback inhibition, significantly redirected carbon flow through the PP pathway and increased lysine production in *Corynebacterium glutamicum* ATCC13032 [28, 29] and riboflavin production in *Bacillus subtilis* RH33 [30]. To test the relationship between availability of NADPH and succinate production, plasmid pZY02E containing *E. coli* genes *zwf*-*gnd* and pZY02 containing deregulated genes *zwf243*-*gnd361* from *C. glutamicum* under the control of inducible promoters P*trc* were constructed and transformed into a succinate producing strain ZTK (W1485, *ΔptsG ldhA pflB*) [31], creating strains ZTK (pZY02E) and ZTK (pZY02). Succinate yield by the resultant strain ZTK (pZY02E), at 1.12 mol/mol glucose, was 11 % higher than the control strain ZTK. However, the specific growth rate and specific glucose uptake rate decreased by 50 and 34 %, respectively. The intracellular metabolite analysis revealed that by-product acetate level was 15 % higher than ZTK (Table 1). As for strain ZTK (pZY02), the molar succinate yield reached the value of 1.18 mol/mol glucose, which was 17 % higher than that of strain ZTK and 5 % higher than that of strain ZTK (pZY02E), and the by-product acetate level was modestly reduced relative to the strain ZTK (pZY02E). The specific growth rate and specific glucose uptake rate decreased by 50 and 25 % compared to the corresponding measurements for ZTK, while the specific glucose uptake rate of ZTK (pZY02) improved by 14 % relative to the strain ZTK (pZY02E) (Table 1). Furthermore, an elevated generation of NADH was favorable for the utilization of deregulated *zwf243*-*gnd36*1, the intracellular NADH/NAD^+^ ratio in ZTK (pZY02), at 0.46, was higher than the ratio 0.26 in ZTK and 0.36 in ZTK (pZY02E) (Fig. 2). It is evident from this experiment that increasing expression of *zwf* and *gnd* was positively correlated with the succinate yield, and recruiting deregulated *zwf243* and *gnd361* were more efficient to achieve a high succinate yield with significantly increased intracellular NADH availability.

**Table 1:** Metabolic profiles of *E.coli* mutants cultivated in NBS medium supplemented with 10 g/L glucose under anaerobic condition

Strains	Fermentation time (h)	CDW (g/L)	Glucose consumed (mM)	Succinate (mM)	Succinate yield (mol/mol)	Acetate (mM)	Pyruvate (mM)	Lactate (mM)	Specific growth rate during 0–24 h (g/g·h)	Specific glucose uptake rate during 0–24 h (g/g·h)
ZTK	42	1.03 ± 0.07	54.26 ± 0.26	54.91 ± 0.29	1.01 ± 0.006	26.10 ± 1.49	–	–	0.042 ± 0.001	0.44 ± 0.01
ZTK (pZY02E)	84	0.75 ± 0.03	53.33 ± 0.45	59.56 ± 0.40	1.12 ± 0.004	30.1 ± 0.03	–	–	0.021 ± 0.002	0.29 ± 0.01
ZTK (pZY02)	72	0.79 ± 0.01	54.26 ± 0.26	64.08 ± 0.40	1.18 ± 0.006	28.18 ± 0.18	–	–	0.021 ± 0.001	0.33 ± 0.03
WSA110	48	0.85 ± 0.01	53.89 ± 0.45	62.40 ± 0.38	1.16 ± 0.005	32.53 ± 0.62	1.50 ± 0.04	–	0.033 ± 0.0004	0.39 ± 0.03
WSA134	48	0.83 ± 0.003	54.26 ± 0.26	65.80 ± 0.22	1.21 ± 0.002	31.48 ± 0.57	–	–	0.029 ± 0.001	0.37 ± 0.006
WSA138	48	0.93 ± 0.008	54.44 ± 0.45	67.74 ± 0.36	1.24 ± 0.005	27.78 ± 0.56	–	–	0.035 ± 0.0008	0.43 ± 0.01
WSA146	48	0.89 ± 0.05	53.33 ± 0.45	69.68 ± 0.26	1.31 ± 0.005	23.68 ± 0.28	–	–	0.032 ± 0.0007	0.40 ± 0.01
WSA150	54	0.76 ± 0.008	51.48 ± 1.31	69.99 ± 1.55	1.36 ± 0.007	3.53 ± 0.04	1.44 ± 0.02	0.78 ± 0.06	0.031 ± 0.002	0.34 ± 0.03
WSA152	48	0.75 ± 0.009	50.93 ± 0.26	66.75 ± 0.35	1.31 ± 0.001	1.74 ± 0.04	3.49 ± 0.11	0.49 ± 0.05	0.023 ± 0.002	0.38 ± 0.02
WSA157	42	0.81 ± 0.014	51.67 ± 1.20	72.50 ± 1.59	1.40 ± 0.002	0.99 ± 0.03	1.71 ± 0.06	0.53 ± 0.09	0.032 ± 0.002	0.41 ± 0.01
WSA159	48	0.69 ± 0.005	52.59 ± 0.26	80.56 ± 0.10	1.53 ± 0.006	1.36 ± 0.14	1.43 ± 0.04	0.85 ± 0.07	0.027 ± 0.002	0.39 ± 0.03
WSA161	36	0.97 ± 0.02	50.56 ± 0.45	73.61 ± 0.70	1.46 ± 0.01	0.97 ± 0.17	0.96 ± 0.09	0.30 ± 0.03	0.051 ± 0.002	0.44 ± 0.03
WSA163	60	0.72 ± 0.05	52.50 ± 0.26	80.72 ± 0.24	1.54 ± 0.01	1.34 ± 0.03	1.51 ± 0.02	0.96 ± 0.02	0.024 ± 0.005	0.28 ± 0.03
WSA165	36	0.90 ± 0.03	52.59 ± 0.52	78.64 ± 0.79	1.50 ± 0.005	2.08 ± 0.05	2.00 ± 0.20	–	0.044 ± 0.002	0.44 ± 0.02
WSA167	36	1.03 ± 0.02	53.33 ± 0.45	77.82 ± 0.55	1.46 ± 0.008	1.87 ± 0.06	2.66 ± 0.14	–	0.054 ± 0.002	0.54 ± 0.005

Bacteria were cultivated with 50 mL NBS medium in a 100 mL flask shaking at 220 rpm and 37 °C in anaerobic conditions with an initial OD600 of 0.6; “–” represents data were not measured in this work. Data are average values and standard deviations of triplicate experiments

**Fig. 3:**
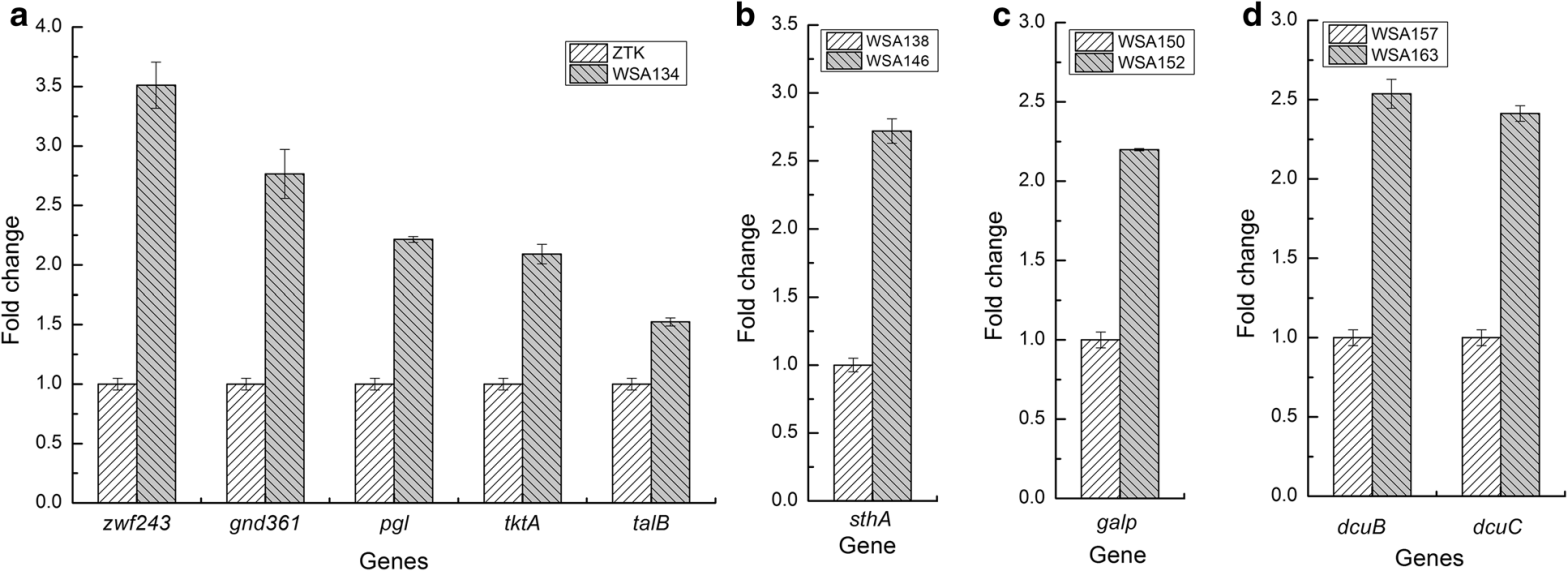
Results of relative transcriptional level. **a** The transcription level of the genes in PP pathway; **b** The transcription level of glucose uptake gene; **c** The transcription level of soluble transhydrogenase gene; **d** The transcription level of the genes in succinate export. Data are average values and standard deviations of triplicate experiments

However, plasmid overexpression has several disadvantages for engineering of genetically stable strains. Plasmid maintenance was a metabolic burden on the host cell, especially for high-copy number plasmids. In addition, only low-copy number plasmids had replication that was timed with the cell cycle, thus, it was difficult to maintain consistent copy number in all cells [13]. Taking these factors into account, we decided to implement all modulation of genes expression directly in chromosome in this study. We subsequently implemented *zwf243* and *gnd*361 expression in chromosome of ZTK by replacing native *lacZ* encoding beta-D-galactosidase, resulting in strain WSA110. *Zwf243* and *gnd361* transcripts were up-regulated by 3.51 and 2.77-folds respectively under the inducible promoter P_*trc*_ (Fig. 3). Succinate yield of this newly constructed strain increased to 1.16 mol/mol glucose, representing 15 % increase relative to the control strain ZTK. The intracellular NADH/NAD^+^ ratio in WSA110 was increased to 0.44 from 0.28 in ZTK (Fig. 2). While addition of IPTG (1 mM) dropped the specific growth rate and specific glucose uptake rate by 21 and 11 %, respectively. The main byproduct acetate was accumulated to 32.53 mM, which was higher than that of strain ZTK. In addition, a small amount of pyruvate had been detected (Table 1).

In order to direct more carbon flow to PP pathway and further redirect flux to glycolic pathway, three critical enzymes of the PP pathway, 6-phosphogluconolactonase (encoded by *pgl*), transketolase (encoded by *tktA*) and transaldolase (encoded by *talB*) were overexpressed in WSA110 by replacing native promoter with constitutive promoter BJ23100 (http://www.parts.igem.org/Part:BBa_J23100). The transcript levels of genes *pgl*, *tktA*, *talB* were up-regulated by 2.2, 2.09, 1.52 folds respectively (Fig. 3). The intracellular NADH/NAD^+^ ratio in WSA134 was elevated to 0.50 (Fig. 2). As expected, succinate yield by the WSA134 was further increased, and the molar yield reached value of 1.21 mol/mol glucose, increasing by 20 % in comparison with strain ZTK and representing 71 % of the maximum stoichiometric yield. Meanwhile, the acetate level slightly reduced to 31.48 mM. The specific growth rate and specific glucose uptake rate of strain WSA134 were decreased by 12 and 5 % (Table 1).

### Effect of overexpression of *pepck* from *A. succinogenes* and conversion of NADPH into NADH on succinate yield

**Fig. 4:**
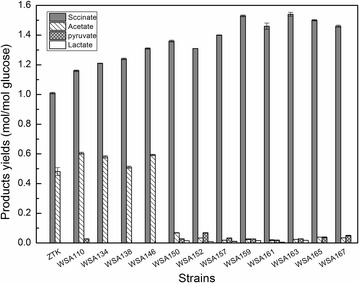
Comparison of the products yields of different mutants. Bacteria were cultivated with 50 mL NBS medium in a 100 mL flask shaking at 220 rpm and 37 °C in anaerobic conditions with an initial OD600 of 0.6. Data are average values and standard deviations of triplicate experiments

The succinate yield was increased by 20 % thanks to modification of the PP pathway, while the acetate production showed a small increase and maintained in a higher level (Fig. 4). Diverting the flux towards succinate by ATP-forming phosphoenolpyruvate carboxykinase (PEPCK) should eliminate the need for the ATP producing acetate pathway, and thus could be used to further improve succinate formation [9]. Therefore, *pepck* (encoding PEPCK) from *A. succinogenes* was heterogenously expressed under the strong constitutive promoter BJ23100 by replacing native *ppc*. In response to this modification, cell concentration of resultant strain WSA138 increased by 12 %, and the specific growth rate and specific glucose uptake rate increased by 21 and 16 % in the anaerobic fermentation. While, the succinate yield increased slightly to 1.24 mol/mol glucose. Correspondingly, the titer of the main byproduct acetate reduced to 27.78 mM (Table 1). We speculated that excess NADPH produced by PP pathway was not transformed into NADH fast enough, thus insufficient NADH supply still limited succinate synthesis in WSA138. Overexpression of transhydrogenase SthA might be able to reinforce intracellular NADH level. In light of this speculation, the native promoter of s*thA* was replaced by the strong constitutive promoter BJ23100 in strain WSA138 to produce strain WSA146. The transcript levels of gene *sthA* was up-regulated by 2.72-folds (Fig. 3). As expected, the intracellular NADH/NAD^+^ ratio was distinctly increased to 0.76 from 0.45 in WSA138 (Fig. 2). The succinate yield increased to 1.31 mol/mol glucose, which represents 76 % of the maximum stoichiometric yield. However, the formation of the main byproduct acetate decreased to 23.68 mM (Table 1).

### Deletion of the *ackA*-*pta* gene leads to increased succinate yield at expense of reduced glucose consumption

Compared to control ZTK, a 30 % increase in terms of succinate yield was achieved by activating PP pathway, overexpressing *sthA* and employing PEPCK as the primary CO_2_-fixation enzyme (Table 2), whereas acetate was always found to be the major byproduct. The presence of acetate at high concentrations is undesirable in the fermentation broth since it interferes with the target succinate biosynthesis and increases the cost of succinate purification [32]. Thus, it is essential to remove acetate formation. Phosphotransacetylase (PTA) and acetate kinase (ACKA), encoded by the *pta* and *ackA* genes respectively, are the major players in acetate synthesis under oxygen deprivation [15]. Deletion of *ackA*-*pta* resulted in strain WSA150, of which acetate production decreased about sixfold and succinate yield increased to 1.36 mol/mol glucose with trace amount of lactate and pyruvate being produced (Table 1). Unfortunately, this yield was obtained at the expense of reduction in specific glucose uptake rate and biomass concentration, each representing 15 and 15 % decrease compared to strain WSA146 (Table 1).

**Table 2:** Comparison of molar yields of succinate in anaerobic fermentation

Strains	Yield of succinate (mol/mol)	Increased yield compared with ZTK (%)	Yield compared with the theoretical maximum yield (%)
ZTK	1.01 ± 0.006	0	59
WSA134	1.21 ± 0.002	20	71
WSA146	1.31 ± 0.005	30	76
WSA157	1.40 ± 0.002	39	82
WSA159	1.53 ± 0.006	51	89
WSA163	1.54 ± 0.010	52	90
WSA165	1.50 ± 0.005	49	88

Bacteria were cultivated with 50 mL NBS medium in a 100 mL flask shaking at 220 rpm and 37 °C in anaerobic conditions with an initial OD600 of 0.6. Data are average values and standard deviations of triplicate experiments

### Overexpressing *galp* gene increases glucose consumption with compromising succinate yield

Disruption of *ackA*-*pta* greatly reduced acetate synthesis, but the mutant strain cultured in minimal medium exhibited low growth and glucose consumption rates. Galactose permease (encoded by *galp*) of *E. coli* was engineered to recover glucose uptake capacity and increase succinate productivity as previously reported [13, 33]. In this work, *galp* was carried out in chromosome with substitution of strong constitutive promoter BJ23100 for native promoter to improve anaerobic glucose transport velocity. The transcript level of gene *galp* was up-regulated by 2.2 folds (Fig. 3). The resultant strain WSA152 was characterized by reduced fermentation time, and its specific glucose uptake rate increased by 12 %. The specific growth rate of WSA152 had a modest reduction, demonstrating that WSA152 had an advantage over WSA150 in terms of individual cell glucose consumed rate. Nevertheless, the succinate yield decreased to 1.31 mol/mol glucose and byproduct pyruvate increased about 1.4-fold to 3.49 mM (Table 1). Moreover, the fermentation broth accumulated little amounts of acetate and lactate. The reasons for this phenomenon may be attributed to that the intracellular glucose input flux is not efficiently channeled into succinate, and in turn leads to increased cell overflow metabolism after *galp* overexpression.

### Overexpressing *pyc* from *C. glutamicum* enhances glucose consumption and succinate yield

The reaction catalyzed by pyruvate carboxylase (PYC) functions as anaplerotic pathways for replenishing oxaloacetate (OAA) under anaerobic conditions [10]. Recruiting heterologous PYC is beneficial to suppress pyruvate formation and further improve succinate yield [34]. Herein, *pyc* encoding pyruvate carboxylase from *C. glutamicum* was introduced into WSA152 by replacing native *lldD* encoding L-lactate dehydrogenase. As expected, the succinate yield elevated to 1.40 mol/mol glucose (amounting to 82 % of the maximum stoichiometric yield) with reducing byproduct pyruvate by half. In addition, trace amount of lactate and acetate were detected in the fermentation broth. Notably, the specific growth rate increased by 39 % and specific glucose uptake rate increased by 8 % in response to PYC recruit (Table 1). The higher succinate yield of WSA157 resulted from the combined effects of increased glucose consumption and suppressed pyruvate production due to an alternative route to OAA pool provided by *pyc* introduction.

### Overexpressing *dcuB* and *dcuC* genes to improve succinate yield close to the stoichiometric maximum yield

*Escherichia coli* contains four Dcu carriers (encoded by *dcuA*, *dcuB*, *dcuC*, and *dcuD* genes) for the transportation of C4-dicarboxylates (succinate, fumarate, and malate) under anaerobic conditions [35, 36]. The uptake, antiport, and export of C4-dicarboxylates are mediated by the Dcu transporters [37]. As decreasing intracellular succinate is an effective strategy for increasing succinate production, we additionally targeted to activate anaerobic C4-dicarboxylates efflux transportation to maximize succinate yield. Therefore, upstream regulated region of the *dcuB* and *dcuC* operons in WSA157 were replaced with strong constitutive promoter BJ23100, respectively, resulting in strains WSA159 and WSA161. Succinate production by the strain WSA159 increased to 80.56 mM, and the molar yield reached the value of 1.53 mol/mol glucose, which is 89 % of the strain-specific stoichiometric maximum. Correspondingly, concomitant decrease in cell growth and glucose consumption was observed for WSA159. Its specific growth rate decreased by 16 % and specific glucose uptake rate decreased by 5 % compared to WSA157 (Table 1). For strain WSA161, this modification increased succinate yield to 1.46 mol/mol glucose, amounting to 85 % of stoichiometric maximum yield. It is worth noting that *dcuC* overexpression increased the specific growth rate by 59 % and specific glucose uptake rate by 7 % over the corresponding measurements for WSA157 (Table 1). These results demonstrated that activation of Dcu transportations effectively accelerated the anaerobic succinate transport out of *E. coli*.

In order to investigate whether the activation of both DcuB and DcuC had a synergistic effect in improving succinate yield, genes *dcuB* and *dcuC* were simultaneously overexpressed in strain WSA157, creating strain WSA163. The transcript levels of genes *dcuB and dcuC* of were up-regulated by 2.54 and 2.41-folds respectively (Fig. 3). The strain WSA163 synthesized succinate with a molar yield of 1.54 mol/mol glucose (90 % of corresponding stoichiometric maximum), which was similar to that of strain WSA159. Nevertheless, the specific growth rate of WSA163 decreased by 25 % and specific glucose uptake rate decreased by 32 % compared to the parent strain WSA157 (Table 1). The intracellular NADH/NAD^+^ ratio in WSA163 was modestly raised (Fig. 2).

### Deletion of the repressor protein binding site leads to removed IPTG induction

In the present work, all genes overexpression was modulated by constitutive promoter BJ23100 with the exception of genes *zwf243* and *gnd361*, which were modulated by the IPTG-induced *trc* promoter. It is not feasible for large-scale production of bulk chemicals and biomass growth with the IPTG induced expression. The repressor protein binding site (AATTGTGAGCGGATAACAATT) was deleted in the two high succinate yield strains WSA159 and WSA163, resulting in strains WSA165 and WSA167, respectively. It was found that deletion the repressor protein binding site dramatically improved final cell concentration. The specific growth rate and specific glucose uptake rate of WSA165 increased by 63 and 13 %, while the succinate yield decreased slightly and maintained at 1.50 mol/mol glucose, which represents 88 % of the maximum stoichiometric yield. The intracellular NADH/NAD^+^ ratio of strain WSA165 still maintained at a high level (Fig. 2). Moreover, this deletion increased the specific growth rate of WSA167 more than one fold and specific glucose uptake rate by 93 % over the corresponding measurements under oxygen deprivation, while its succinate yield decreased to 1.46 mol/mol glucose. In addition, a small amount of pyruvate and acetate had been detected in the media of both strains (Table 1).

## Discussion

Succinate synthesis through the reductive branch of the TCA cycle with the CO_2_ fixation is an attractive option under anaerobic conditions [4, 24]. However, the maximum theoretical yield of succinate through the glycolysis and reductive TCA cycle is 1 mol/mol glucose resulting from the NADH limitation [14, 24, 38]. It is well known that the excess NADH would upset the flux distribution among the end products: succinate, acetate and ethanol [9]. There is no excess NADH to be oxidized by producing lactate or ethanol in the ZTK strain (W1485, *ΔptsG ldhA pflB*), while NADH supply is still limited for higher yield succinate production. In this work, PP pathway was verified for the first time to provide more NADH for succinate production compared with the traditional glycolysis pathway. On the premise that NADPH produced by PP pathway can be immediately converted into NADH through transhydrogenase SthA, the stoichiometric maximum succinate yield of 1.714 mol/mol glucose can be obtained if the carbon flux ratio between PP pathway and glycolysis is 6:1. Based on this theoretical guidance, we deregulated the PP pathway and glycolytic pathway flux ratio and further strengthened the conversion of NADPH into NADH. We systematically adjusted the expression of five critical enzymes of PP pathway for succinate production. The deregulated genes *zwf243* and *gnd361* involved in NADPH generation from *C. glutamicum* were firstly introduced into *E. coli* for succinate production for the first time. We compared the effects of overexpression of native *zwf*-*gnd* and deregulated *zwf243*-*gnd361* on succinate production and NADH/NAD^+^ ratio. It was found that co-expression of the mutated dehydrogenases, which removed allosteric inhibition by intracellular metabolites, was more efficient to achieve a high succinate yield with significantly increased intracellular NADH availability. An improvement by 15 % of the succinate yield was obtained by overexpressing of the deregulated *zwf243* and *gnd361* directly in chromosome of ZTK. In this study, increasing carbon flux towards PP pathway through expression of deregulated *zwf243* and *gnd361* was investigated in strain ZTK (W1485, *ΔptsG ldhA pflB*), and based on this genetic background, high-yield succinate production in strain ZTK was restricted by multiple limiting factors, including NADH availability, carbon flux towards succinate, succinate export, and etc. It is reasonable that only a minor contribution to the succinate yield improvement (increased by 15 %) by introduction of deregulated *zwf243* and *gnd361* into strain ZTK. An extended application of mutant *zwf243* and *gnd361* is still recommended for the production of products directly stemming from the PP pathway as well as other NADH-demanding compounds in *E. coli*. Three critical genes of PP pathway were then overexpressed to further divert more carbon flux to the PP pathway. The engineered strain WSA134 by disturbing PP pathway achieved succinate yield of 1.21 mol/mol glucose, increased 20 % compared to ZTK, amounting to 71 % of theoretical maximum yield (Table 2). These results indicated that more carbon flux went through the PP pathway, thus leading to production of more reducing equivalent in the form of NADPH, which was then converted into NADH through transhydrogenase SthA for succinate synthesis.

In addition, for the production of succinate, one of the key factors is the supply of CO_2_ [4]. If all carbon flux goes through glycolysis/reductive TCA cycle, the maximum theoretical yield of succinate resulting from the NADH limitation is based on the reaction: C_6_H_12_O_6_ + CO_2_ → C_4_H_6_O_4_ + H_2_O, in this case one CO_2_ is required for the synthesis of one succinate. By contrast, if 1/7 carbon flux goes through glycolysis/reductive TCA cycle and 6/7 carbon flux goes through the combined PP pathway and glycolysis/reductive TCA route to meet the NADH requirement, the maximum theoretical yield of succinate is based on the stoichiometry of the following redox reaction: C_6_H_12_O_6_ + 0.857 CO_2_ → 1.714 C_4_H_6_O_4_ + 0.857 H_2_O, so in this case only 0.5 (0.857/1.714) CO_2_ is required for the synthesis of one succinate. Apparently, less exogenous CO_2_ is required for succinate synthesis using PP pathway.

Carbon fluxes at the PEP node serve to limit the amount of succinate produced for redox balance during the anaerobic glucose fermentation. Besides, these fluxes must provide sufficient energy (ATP) for growth, maintenance, and precursors for succinate biosynthesis [11]. In this work, we carried out a theoretical metabolic flux balance analysis (FBA) [39] of succinate production by *E. coli* under anaerobic conditions with/without *ppc* replacement by *pepck*. Flux distributions computed by FBA (data not shown) showed that sole production of succinate through the reductive TCA cycle does not lead to ATP generation without *ppc* replacement by *pepck*, ATP production is mainly associated with acetate formation, but the presence of acetate at high concentrations is undesirable in the fermentation broth since it interferes with the target succinate biosynthesis and increases the cost of succinate purification [32]. When native *ppc* is replaced by *A. succinogenes pepck*, the conversion of glucose to succinate will lead to net production of ATP. With the presence of PEPCK, the ATP production associated with succinate production corresponding to the maximum theoretical yield of succinate is based on the reaction: C_6_H_12_O_6_ + 0.857 CO_2_ → 1.714 C_4_H_6_O_4_ + 1.714 ATP + 0.857 H_2_O, the conversion of 1 mol glucose to succinate will produce 1.714 mol ATP. Diverting the flux towards succinate by ATP-forming PEPCK should increase carbon flow into the four-carbon intermediate OAA for succinate production, increase the net production of ATP for growth and maintenance and eliminate the need for the ATP producing acetate pathway [11]. In our existing work, *pepck* overexpression indeed increased the specific growth rate, specific glucose uptake rate and biomass by 21, 16 and 12 %, respectively, and acetate level decreased from 31.48 to 27.78 mM (Table 1). While this modification did not result in significant increase in succinate yield, this might be because excess NADPH was not converted into NADH fast enough. The succinate yield was further increased through *sthA* overexpression. Apparently, transhydrogenase SthA plays an important role in terms of NADH supply. It should be a potential strategy to regulate the NADH generation for succinate synthesis through combining action of the PP pathway and transhydrogenase SthA.

Deletion *ackA*-*pta* promoted succinate yield but decreased the cells’ overall fitness. Heterologous expression of PYC from *C. glutamicum* led to higher succinate yield, and restored the ability of the engineered strain to ferment glucose. 90 % of stoichiometric maximum succinate yield was achieved after modulating succinate efflux transportation. The deletions of repressor protein binding sequences led to the constitutive expression of the PP pathway genes *zwf243*-*gnd361*, and the engineered strains were observed to increase cellular fitness during anaerobic fermentation. Future engineering efforts could focus on modulating the delicate split of the carbon flux between PP pathway and glycolytic pathway, thus leading to achieve succinate yield close to the target value of 1.714 mol/mol glucose.

## Conclusions

For the first time, pentose phosphate pathway was rationally designed to improve NADH supply for efficient succinate yield in *E. coli* under anaerobic conditions. Through further combing diverting flux towards succinate and engineering its export, the succinate yield reached the value of 1.54 mol/mol glucose, representing 90 % of stoichiometric maximum. The systems metabolic engineering strategy described in this work may also be useful for the synthesis of other related NADH-demanding products.

## Methods

### Bacterial strains, primers, and media

Bacterial strains and plasmids used in this study were listed in Table 3. All the primers used in this study were listed in Additional file 1: Table S1. *E. coli* ZTK (W1485; *ΔptsG ΔldhA ΔpflB*) [31] was used as the parent strain for construction of all mutants described in this study. *E. coli* DH5α was used as the host for plasmid construction. *E. coli* cells were cultivated at 37 or 30 °C in a rotatory shaker at 220 rpm in Luria–Bertani (LB). Ampicillin (100 µg/mL), tetracycline (100 µg/mL), spectinomycin (100 µg/mL) and chloramphenicol (16 µg/mL) were added as needed.

**Table 3:** Strains and plasmids used in this study

Strains and plasmids	Relevant characteristics	Sources or references
Strains		
ZTK	W1485, *ΔptsG ΔldhA ΔpflB*; kan^r^	Lab stock
WSA110	ZTK, *ΔlacZ::*P_*trc*_-*zwf243*-*gnd361* from *C. glutamicum*; kan^r^	This study
WSA134	WSA110, P_*BJ23100*_-*talB*; P_*BJ23100*_-*tktA*; P_*BJ23100*_-*pgl*; kan^r^	This study
WSA138	WSA134, P_*BJ23100*_-*pepck*; *Δppc::*P_*BJ23100*_-*pepck* from *A. succinogenes*; kan^r^	This study
WSA146	WSA138, P_*BJ23100*_-*sthA*; kan^r^	This study
WSA150	WSA146, *ΔackA*-*pta*; kan^r^	This study
WSA152	WSA150, P_*BJ23100*_-*galp*; kan^r^	This study
WSA157	WSA152, *ΔlldD::*P_*BJ23100*_-*pyc* from *C. glutamicum*; kan^r^	This study
WSA159	WSA157, P_*BJ23100*_-*dcuB*; kan^r^	This study
WSA161	WSA157, P_*BJ23100*_-*dcuC*; kan^r^	This study
WSA163	WSA157, P_*BJ23100*_-*dcuB*; P_*BJ23100*_-*dcuC*; kan^r^	This study
WSA165	WSA159, *Δzwf243*-*gnd361*-*protein bind*; kan^r^	This study
WSA167	WSA163, *Δzwf243*-*gnd361*-*protein bind*; kan^r^	This study
DH5α	Cloning host	Lab stock
Plasmids		
pZY02E	p15 ori; P*trc*-*zwf*-*gnd* of *E.coli*; Cm^r^	This study
pZY02	p15 ori; P*trc*-*zwf243*-*gnd361* of *C. glutamicum*; Cm^r^	Lab stock
pCU18	pBR322 ori; Amp^r^	Lab stock
pCU18-*zwf243*-*gnd361*	P*trc*-*zwf243*-*gnd361* from pZY02, *cat* gene from pEL04, upstream and downstream fragments of *lacz* gene were cloned into pCU18; Amp^r^, Cm^r^	This study
pCU18-*pyc*	P_*BJ23100*-_ *pyc* from *C. glutamicum* was cloned into pCU18; Amp^r^	This study
pCU18-*ppc*	Upstream and downstream fragments of *ppc* gene were cloned into pCU18; Amp^r^	This study
pCU18-*cat*-*sacB*	*cat*-*sacB* genes from pEL04 were cloned into pCU18-*ppc*; Amp^r^, Cm^r^	This study
pCU18-*pepck*	P_*BJ23100*-_ *pepck* from *A. succinogenes* was cloned into pCU18-*ppc*; Amp^r^	This study
pEL04	*cat*-*sacB* cassette; Cm^r^	[41]
PTKSS	p15A replication, Tet^r^, I-SceI restriction sites; Cm^r^, Tet^r^	[49]
PTKRED	pSC10 replication, temperature sensitive replication origin, ParaBAD-driven *I*-*SceI* gene, red recombinase expression plasmid, lac-inducible expression; Spc^r^	[49]

*kan* kanamycin; *Amp* ampicillin; *Cm* chloramphenicol; *Tet* tetracycline; *Spc* spectinomycin; *r* resistance

### Plasmid construction

To construct pZY02E, *zwf*, *gnd* genes from *E. coli* with the synthesized ribosome binding sites (RBSs) were amplified by polymerase chain reaction (PCR) using the primers *pzw*-*F/pzw*-*R*, *pgd*-*F/pgd*-*R* respectively. Primers *pzy*-*F/pzy*-*R* were used to amplify the backbone of the plasmid pZY02. The *zwf*-*gnd* genes of *E. coli* were cloned into pZY02 by replacing *zwf243*-*gnd361* genes to yield pZY02E. The method used for the construction of pZY02E was based on the utilization of circular polymerase extension cloning (CPEC) [40].

To construct pCU18-*zwf243*-*gnd361*, upstream and downstream fragments of *lacz* gene were amplified from *E. coli* genome using primers *lacz*-*up*-*F*/*lacz*-*up*-*R* and *lacz*-*down*-*F*/*lacz*-*down*-*R*, *cat* gene and *zwf243*-*gnd361* genes were amplified from pEL04 [41] and pZY02 (containing *zwf243* and *gnd361* mutation under *trc* promoter) using primers *cm*-*F/cm*-*R* and *zw*-*F/zw*-*R*, respectively. The upstream and *cat* gene fragments were fused and amplified by fusion PCR with primers *lacz*-*up*-*F*/*cm*-*R,* three fragments were then digested with SmaI-SacI, HindIII-PstI, SalI-SmaI respectively and ligated into pCU18 digested with the same enzymes to create pCU18-*zwf243*-*gnd361*.

For the construction of pCU18-*pyc*, the fragments of *pyc* gene with promoter and ribosomal binding site of *E. coli* was amplified from *C. glutamicum* genome using primers *pyc*-*F*/*pyc*-*R* and digested with SphI- SacI. The digested fragment was inserted into pCU18 with the same enzymes to yield pCU18-*pyc*.

For pCU18-*cat*-*sacB* construction, the *ppc* and *cat*-*sacB* fragments were amplified from *E. coli* genome and pEL04 using primers *ppc*-*up*-*F/ppc*-*down*-*R* and *cat*-*F/sacB*-*R* respectively. The *ppc* fragment was digested with HindIII- SmaI and ligated into pCU18 digested with the same enzymes to yield pCU18-*ppc*. The obtained plasmid was digested with MIuI-SphI and ligated with *cat*-*sacB* fragment digested with the same restriction enzymes to give plasmid pCU18-*cat*-*sacB*.

For pCU18-*pepck* construction, *pepck* fragments was amplified from *A. succinogenes* genome using primers *pck*-*F/pck*-*R*. The PCR product was digested with MIuI-SphI and inserted into the same sites of pCU18-*ppc* to yield pCU18-*pepck*.

### Strain construction

Chromosomal modifications were performed using the method of λ-red recombination with the corresponding recombinant fragments. The final fragments were transformed into the competent cells with expression of the λ-red recombination enzymes, and the procedure in detail was achieved as described previously [42, 43]. The method used for the integration of *pyc* in the chromosome of *E. coli* was based on the utilization of CRISPR–Cas9 meditated genome editing system [44, 45].

### Anaerobic fermentation

The composition of the NBS medium used for succinate production was achieved as described previously [46]. All fermentations were carried out at 37 °C in a rotatory shaker at 220 rpm. Single bacterial colonies were grown in a 15 ml glass tube containing 5 ml of LB medium for 8 h. The culture was then inoculated into 500 mL flask containing 90 mL LB medium with an initial OD_600_ about 0.05 and then cultured for 5 h. Cells were harvested by centrifugation for 5 min at 6000 rpm and 4 °C, washed once with 50 mL NBS and used as an inoculum for the fermentation. The fermentation was carried out in triplets, in sealed 100-mL bottles containing 50 mL of NBS medium supplement with 10 g/L glucose, 100 mM NaHCO_3_. 1 mM of isopropyl-β-D-thiogalactopyranoside (IPTG) was added at the beginning of anaerobic culture to explore the appropriate expression quantity of mutant genes *zwf*243 and *gnd*361 for succinate production. At the start of the dual-phase fermentation, the cells were grown in the existed small amount of air which keeps a short time of microaerobic condition and was consumed rapidly.

### Analytical techniques

Cell growth was monitored by measuring the OD_600_ with an ultraviolet spectrophotometer (Beijing Puxi Universal Co. Ltd). The biomass concentration was calculated from OD_600_ values using an experimentally determined correlation with 1 OD_600_ unit being equal to 0.38 g/L cell dry weight (CDW) [42]. Glucose in the fermentation broth was determined utilizing a SBA sensor machine (Institute of Microbiology, Shangdong, China). To determine succinate, pyruvate, acetate and lactate concentrations, culture samples were centrifuged at 12,000*g* for 5 min and the aqueous supernatant used for HPLC analysis on an Agilent 1100 Series HPLC system equipped with a cation exchange column. (Aminex HPX87-H, Bio-Rad, Hercules, CA, USA), a UV absorbance detector (Agilent Technologies, G1315D) and a refractive index (RI) detector (Agilent Technologies, HP1047A). A mobile phase of 5 mM H_2_SO_4_ solution at a 0.4 mL/min flow rate was used. The column was operated at 65 °C. The intracellular NADH and NAD^+^ levels were determined by NAD/NADH Quantitation Kit (SIGMA) [47].

### Quantitative real-time reverse transcription (RT)-PCR analysis

*Escherichia coli* ZTK and its derivatives were harvested when grown to the exponential phase under anaerobic condition. Total RNA was extracted with RNAprep pure Kit (Tiangen, Beijing, China). 500 ng of total RNA was transcribed into cDNA using Quant Reverse Transcriptase with random primers (Tiangen, Beijing, China). Samples were then analyzed using a Light Cycler 480 II (Roche, Basel, Switzerland) with Real Master Mix (SYBR Green). The 16S rRNA gene was selected as reference for normalization and three biological replicates were performed. The obtained data were analyzed by using the 2^−ΔΔCt^ method previously described [48].

## Additional file

10.1186/s12934-016-0536-1 Primer sequence used in this study.

## Abbreviations

PP: pentose phosphate
TCA: reductive tricarboxylic acid
GldA: glycerol dehydrogenase
FBP: fructose 1,6-bisphosphate
Gra3P: D-glyceraldehyde 3-phosphate
PEPCK: phosphoenolpyruvate carboxykinase
PTA: phosphotransacetylase
ACKA: acetate kinase
PYC: pyruvate carboxylase
OAA: oxaloacetate
IPTG: isopropyl-β-D-thiogalactopyranoside
CDW: cell dry weight
RI: refractive index
