# Supplementary material for: High-yield anaerobic succinate production by strategically regulating multiple metabolic pathways based on stoichiometric maximum in Escherichia coli
Source: Microb Cell Fact. 2016 Aug 12;15:141. doi: 10.1186/s12934-016-0536-1 (PMC4983090; doi:10.1186/s12934-016-0536-1)
Supplement: Supplementary file 1 — 10.1186/s12934-016-0536-1 Primer sequence used in this study. [file 12934_2016_536_MOESM1_ESM.docx]

**Supplementary Table S1. Primer sequence used in this study**

| Primer name | Primer sequence (5’ 3’) |
| --- | --- |
| *lacz-up-F* | TGACCAGACACCCATCAACA |
| *lacz-up-R* | ATTACCCTGTTATCCCTACGTTATGATGCACATTAATTGCGTTGCGCTCACTG |
| *cm-F* | TGTGCATCATAACGTAGGGATAACAGGGTAATCCTGTGACGGAAGATCACTTCG |
| *cm-R* | AAGCGGCTATTTAACGACCCT |
| *zw-F* | CTGATGTCGACTAGGGATAACAGGGTAATGCGGGCAGTGAGCGCAACGCAATTAATGTGCATCATAACGGTTCTGGCA |
| *zw-R* | ACTTGACCCGGGACTAGGGGTGTTAAGCTTCAACCTCGGAG |
| *lacz-down-F* | ACTTGACCCGGGCATGTCTGCCCGTATTTCGCGT |
| *lacz-down-R* | TGACTTGAGCTCGAAATTACTGCGACGGCTGA |
| *pgl-up-F* | GTTTGCGCCCAGTTAGATGTG |
| *pgl-up-R* | GCTAGCACTGTACCTAGGACTGAGCTAGCCGTCAACTTTTGGCGGTGAATGCTGA |
| *pgl-tet-F* | TTGACGGCTAGCTCAGTCCTAGGTACAGTGCTAGCTAGGGATAACAGGGTAATGTACC |
| *pgl-tet-R* | GCTAGCACTGTACCTAGGACTGAGCTAGCCGTCAAATTACCCTGTTATCCCTACTAAGC |
| *pgl-down-F* | TTGACGGCTAGCTCAGTCCTAGGTACAGTGCTAGCAGCGACTAATTTTAGCTGTTACAG |
| *pgl-down-R* | TGAGATATTGGCGGAATGGCA |
| *pgl-c-F* | CAGGGCTTTAATAACGGGCA |
| *pgl-c-R* | ACAAACTGCCCCTGGTGATC |
| *tkt-up-F* | GTCAGACGTTTAGCGTATTCGCT |
| *tkt-up-R* | GCTAGCACTGTACCTAGGACTGAGCTAGCCGTCAATGCCAACAATTTACCGCAAG |
| *tkt-tet-F* | TTGACGGCTAGCTCAGTCCTAGGTACAGTGCTAGCTAGGGATAACAGGGTAATGTACC |
| *tkt-tet-R* | GCTAGCACTGTACCTAGGACTGAGCTAGCCGTCAAATTACCCTGTTATCCCTACTAAGC |
| *tkt-down-F* | TTGACGGCTAGCTCAGTCCTAGGTACAGTGCTAGCCAAATTTTCCGGCGTAGCCCA |
| *tkt-down-R* | AGGTGTAGTGGTCGACAATGT |
| *tkt-c-F* | GTTTTCACCTGCGCATCACTC |
| *tkt-c-R* | TGCAGCTGACGGAAGTTTTTC |
| *talB-up-F* | CACATCCAAAAGACGGCTCCA |
| *talB-up-R* | GCTAGCACTGTACCTAGGACTGAGCTAGCCGTCAAGCCCTGATGATATCACGACGCA |
| *talB-tet-F* | TTGACGGCTAGCTCAGTCCTAGGTACAGTGCTAGCTAGGGATAACAGGGTAATGTACC |
| *talB-tet-R* | GCTAGCACTGTACCTAGGACTGAGCTAGCCGTCAAATTACCCTGTTATCCCTACTAAGC |
| *talB-down-F* | TTGACGGCTAGCTCAGTCCTAGGTACAGTGCTAGCAGACCGGTTACATCCCCCTAAC |
| *talB-down-R* | CAGTCAAGAATACGGCCAACA |
| *talB-c-F* | CTGGCTGTGGATGAATGCTA |
| *talB-c-R* | CTTTTTCCAGCTGTTCTGCAG |
| *ppc-up-F* | GTAGCAAGCTTGATACTTGCGCATCTTATCCGACCTACACCT |
| *ppc-down-R* | AGTAACCCGGGGAAAACGAGGGTGTTAGAAC |
| *cat-F* | ACTGTACGCGTCCTGTGACGGAAGATCACTTCG |
| *sacB-R* | AGTGTGCATGCCTGAGGTTCTTATGGCTCTTG |
| *pck-F* | ACTGTACGCGTTTGACGGCTAGCTCAGTCCTAGGTACAGTGCTAGCGAATTCCAAGGAGGTGAAGTA |
| *pck-R* | AGTGTGCATGCAGTTTTATGCTTTTGGACCGGCGCCA |
| *pck-up-F* | TCCAGAATCAAAAGGTGGGT |
| *pck-up-R* | CTGTACCTAGGACTGAGCTAGCCGTCAAAGTAAAGTCTTTTTGGGGGTGTTAACCGCGA |
| *pck-tet-F* | TTGACGGCTAGCTCAGTCCTAGGTACAGTGCTAGCTAGGGATAACAGGGTAATGTACC |
| *pck-tet-R* | GCTAGCACTGTACCTAGGACTGAGCTAGCCGTCAAATTACCCTGTTATCCCTACTAAGC |
| *pck-down-F* | ACGGCTAGCTCAGTCCTAGGTACAGTGCTAGCATTCAGGCAATACATATTGGCTAAGGA |
| *pck-down-R* | ACGAACAGACGTTTGCCGGAAAGCT |
| *pck-c-F* | TCCCGCCATATAAACCAAGA |
| *pck-c-R* | CAGAAAGTATCGCGAGTGGT |
| *sthA-up-F* | CACTTCACGCAAACTCAGGCT |
| *sthA-up-R* | CACTGTACCTAGGACTGAGCTAGCCGTCAACTGTTCTTATACATAAAAGCAACAGAATG |
| *sthA-tet-F* | TTGACGGCTAGCTCAGTCCTAGGTACAGTGCTAGCTAGGGATAACAGGGTAATGTACC |
| *sthA-tet-R* | GCTAGCACTGTACCTAGGACTGAGCTAGCCGTCAAATTACCCTGTTATCCCTACTAAGC |
| *sthA-down-F* | GGCTAGCTCAGTCCTAGGTACAGTGCTAGCGTAAGCCCTACCATGCCACATTC |
| *sthA-down-R* | GTTGGATGATATGGACGAGAG |
| *AA-up-F* | TCCGGCGTTGACATGCTTCA |
| *AA-up-R* | CACCATTTACTGCATCGATG |
| *AA-tet-F* | TTGACGGCTAGCTCAGTCCTAGGTACAGTGCTAGCTAGGGATAACAGGGTAATGTACC |
| *AA-tet-R* | GCTAGCACTGTACCTAGGACTGAGCTAGCCGTCAAATTACCCTGTTATCCCTACTAAGC |
| *AA-down-F* | ACTGAAATTTGCCATCATCGATGCAGTAAATGGTGCATCTTCCCGGATCTGAACA |
| *AA-down-R* | ACCTGATACTGCACTTCCTG |
| *AA-c-F* | TCAGGTATCCTTTAGCAGCCT |
| *AA-c-R* | TGGATTCAGTGATTGCGGACA |
| *gp-up-F* | TCTTTCTTCACCTGCGTTCAAAG |
| *gp-up-R* | CTAGCTAGCACTGTACCTAGGACTGAGCTAGCCGTCAAGTGAATTAAGATAGGTGAG |
| *gp-tet-F* | TTGACGGCTAGCTCAGTCCTAGGTACAGTGCTAGCTAGGGATAACAGGGTAATGTACC |
| *gp-tet-R* | GCTAGCACTGTACCTAGGACTGAGCTAGCCGTCAAATTACCCTGTTATCCCTACTAAG |
| *gp-down-F* | GGGTAATTTGACGGCTAGCTCAGTCCTAGGTACAGTGCTAGCAATAAAAAATAACCATAT |
| *gp-down-R* | CAGAAAGATAAGCACCGAGGAT |
| *gp-c-F* | CTTTTGGATAGGCGTTCACGC |
| *gp-c-R* | AGGTACAGCGGTGCGGTATAAG |
| *pyc-F* | ACTGCATGCACTGAAATTTGCCATCATCGATGCAGTAAATGGTGTTGACGGCTAGCTCAGTCCTAGGTACAGTGCTAGCAAAGAGGAGAAAATGTCGACTCACACATCT |
| *pyc-R* | ACTGCGAGCTCATTCT ACTAGTTCTAGA TTAGGAAACGACGACGATCAAGTC |
| *lldD-up-F* | TTCGATATTCTGGAAGCCCGCTAC |
| *lldD-up-R* | TTCGATATTCTGGAAGCCCGCTAC |
| *pyc-up-F* | GATGCAGTAAATGGTGTTGACGGCTAG |
| *pyc-up-R* | TTAGGAAACGACGACGATCAAGTCG |
| *lldD-down-F* | AGGTGGAAGGTGGCGACTTGATCGTCGTCGTTTCCTAATTGCAAGCGGTGACACATCCG |
| *lldD-down-R* | ATTTAGCAGGTTAGCTACACCCGCC |
| *DB-up-F* | CGCTGGTTATCTGTAAGTAA |
| *DB-up-R* | CTAGCTAGCACTGTACCTAGGACTGAGCTAGCCGTCAACCTATTTAAATTTTTGCTGAA |
| *DB-tet-F* | TTGACGGCTAGCTCAGTCCTAGGTACAGTGCTAGCTAGGGATAACAGGGTAAT |
| *DB-tet-R* | GCTAGCACTGTACCTAGGACTGAGCTAGCCGTCAAATTACCCTGTTATCCCTA |
| *DB-down-F* | GTAATTTGACGGCTAGCTCAGTCCTAGGTACAGTGCTAGCATTATCGCGAGGGTTCACACA |
| *DB-down-R* | GAATGGTGATTGCCAGCAGAT |
| *DB-C-F* | TCTCTTCAGGTGAACGGTGTT |
| *DB-C-R* | CATCGGACGTTCCGGACGGAT |
| *DC-up-F* | TCTACTACTAGCATAGCAAAGC |
| *DC-up-R* | CCTAGCTAGCACTGTACCTAGGACTGAGCTAGCCGTCAATTAAGACATTGAAGTTGCTGT |
| *DC-tet-F* | TTGACGGCTAGCTCAGTCCTAGGTACAGTGCTAGCTAGGGATAACAGGGTAAT |
| *DC-tet-R* | GCTAGCACTGTACCTAGGACTGAGCTAGCCGTCAAATTACCCTGTTATCCCTA |
| *DC-down-F* | AATTTGACGGCTAGCTCAGTCCTAGGTACAGTGCTAGCTGGCAGTTTTTCTTGATTTTAATC |
| *DC-down-R* | ATCACCGGAAATAGGGTTGCCA |
| *DC-C-F* | GTTAAATATTTCCGCGCATCG |
| *DC-C-R* | TAAGCGGCAAATCCACACAGC |
| *pro-up-F* | CAAATGCTGAATGAGGGCATC |
| *pro-up-R* | CCACACATTATACGAGCCGGATGATTAATTGTCAAC |
| *pro-tet-F* | GTTGACAATTAATCATCCGGCTCGTATAATGTGTGGTAGGGATAACAGGGTAAT |
| *pro-tet-R* | CCACACATTATACGAGCCGGATGATTAATTGTCAACATTACCCTGTTATCCCTA |
| *pro-down-F* | GTTGACAATTAATCATCCGGCTCGTATAATGTGTGGTCACACAGAATTCGACTCGGT |
| *pro-down-R* | CTGGTGGCAGACCGCTGTGAAG |
| *pro-c-F* | TGGTGCGGATATCTCGGTAG |
| *pro-c-R* | GAGGTTGTCGAAAGCTGCATC |
| *T1* | GCGTGAAGTGGTTCGGTTG |
| *T2* | CTGTAATGCAGGTAAAGCGATC |
| *Pzw-F* | ATGATGGTACCCGATACTACTTTTACATTAAGGAGGTTAACAGATGGCGGTAACGCAAACAGCCCA |
| *Pzw-R* | TTACTCAAACTCATTCCAGGAACGACCATCACG |
| *Pgd-F* | CGTGATGGTCGTTCCTGGAATGAGTTTGAGTAAGCCGACCAAGGATAAGGAGATAATATATGTCCAAGCAACAGATCGGCGT |
| *Pgd-R* | TTAATCCAGCCATTCGGTATGGAACACACCTTC |
| *Pzy-F* | AAGAAGGTGTGTTCCATACCGAATGGCTGGATTAAATGAAGCAAACAGTTTATATC |
| *Pzy-R* | CTCCTTAATGTAAAAGTAGTATCGGGTACCATCATTGTTAACCTCCTTAATGTAAAAG |
